# The Road to HTLV-1-Induced Leukemia by Following the Subcellular Localization of HTLV-1-Encoded HBZ Protein

**DOI:** 10.3389/fimmu.2022.940131

**Published:** 2022-06-23

**Authors:** Roberto S. Accolla

**Affiliations:** Laboratories of General Pathology and Immunology “Giovanna Tosi”, Department of Medicine and Surgery, University of Insubria, Varese, Italy

**Keywords:** HTLV-1, HBZ, ATL, HAM/TSP, Tax-1, human retrovirus

## Abstract

Human T cell leukemia virus-1 (HTLV-1) is the causative agent of a severe cancer of the lymphoid lineage that develops in 3-5% of infected individuals after many years. HTLV-1 infection may also induce a serious inflammatory pathology of the nervous system designated HTLV-associated myelopathy/tropical spastic paraparesis (HAM/TSP). Two virus-encoded proteins, the viral transactivator Tax-1 and the HTLV-1 basic leucine-zipper factor HBZ, are strongly involved in the oncogenic process. Tax-1 is involved in initial phases of the oncogenic process. Conversely, HBZ seems to be involved in maintenance of the neoplastic state as witnessed by the generation of leukemic/lymphomatous phenotype in HBZ transgenic mice and the persistent expression of HBZ in all phases of the oncogenic process. Nevertheless, the intimate molecular and cellular mechanism mediated by the two viral proteins, particularly HBZ, in oncogenesis still remain elusive. An important step toward the complete comprehension of HBZ-associated oncogenicity is the clarification of the anatomical correlates of HBZ during the various phases of HTLV-1 infection to development of HTLV-1-associated inflammatory pathology and ultimately to the establishment of leukemia. In this review, I will summarize recent studies that have established for the first time a temporal and unidirectional expression of HBZ, beginning with an exclusive cytoplasmic localization in infected asymptomatic individuals and in HAM/TSP patients and ending to a progressive cytoplasmic-to-nuclear transition in leukemic cells. These results are framed within the present knowledge of HTLV-1 infection and the future lines of research that may shed new light on the complex mechanism of HTLV-1- mediated oncogenesis.

## Introduction

The human T cell leukemia/lymphoma virus-type 1 (HTLV-1), the first described human retrovirus (1) is the etiological agent of a very severe form of cancer of the lymphoid lineage designated Adult T cell leukemia (ATL) (2). HTLV-1 infects at least 10-15 million people worldwide (3) and this number is probably underestimated because of the lack of epidemiological studies in various and highly populated areas of the world. The virus is highly endemic in South Japan, central Africa, Iran, the Caribbean islands and central Australia (4). Infection is transmitted vertically by breast feeding from infected mothers to neonates and horizontally by sexual intercourse and blood transfusion (5). Unlike HIV-1 infection, HTLV-1 infection is transmitted by cell-to-cell contact. From a pathological viewpoint, HTLV-1 infection can remain silent lifelong in the vast majority of individuals. However in 5 to 7% of infected people infection can generate serious forms of chronic inflammatory diseases including a severe form of neuroinflammation designated HTLV-1-associated myelopathy/tropical spastic paraparesis (HAM/TSP) (6–8) and, as mentioned above, the clinically still untreatable ATL. Considering the high percentage of HTLV-1 infected people that progress toward ATL, it appears justified the recent definition of HTLV-1 virus as the most potent “oncogene” ever described at least among the pathogens that have been associated to neoplastic transformation (9). The sense strand of HTLV-1 genome encompasses gag-pol-env genes common to all retroviruses as well as the pX region encoding regulatory proteins of the virus and particularly the Tax-1 protein necessary for the activation of transcription of the viral genome (10). Interestingly, the anti-sense strand of the viral genome contains an open reading frame that was first described in 1989 by Larocca et al. (11). The transcribed product and the corresponding protein designated HTLV-1 basic leucine zipper or HBZ were characterized in 2002 by the group of Jean-Michel Mesnard (12). Here is important to stress that for many years the existence of anti-sense transcripts and corresponding proteins made by retroviruses has been underestimated if not unrecognized. Hopefully the paradigm of “retroviruses encoded proteins on only one strand of proviral DNA” has shifted, mostly because of the discovery of HBZ (13).

HBZ contains a bZIP domain in addition to an activation (N-terminus) and a central domain (12). HBZ is expressed mainly in two distinct isoforms: a spliced form containing 206 amino acids (sp1) and an unspliced form with 209 amino acids (us) (14, 15). The sp1 form is more abundant and is found in almost all ATL patients (16). One of the crucial functions of HBZ, particularly of the sp1 form, resides in inhibiting sense transcription from viral 5’ LTR by interacting with CREB-2 *via* its bZIP domain. This results in strong inhibition of the CREB-2/Tax-1 interaction instrumental for the activation of HTLV-1 LTR (12).

Initial studies mainly performed by using cDNA constructs of the HBZ gene showed that HBZ exerts frequently opposing effects with respect to Tax-1 (17). For example, HBZ inhibits, while Tax-1 activates, the classical Nuclear Factor kappa B (NFkB) pathway by inducing PDLIM2 expression which brings about proteasomal degradation of RelA (18). Similarly HBZ suppresses NFAT and AP-1 pathways, whereas Tax-1 activate them. HBZ suppresses, while Tax-1 activates, Wnt pathway by interacting with the disheveled-associating protein with a high frequency of Leucine residues (DAPLE) (19). On the other hand, HBZ activates the TGF-β/Smad pathway while Tax-1 inhibits it (20).

Tax-1 and HBZ are considered the two crucial factors for the oncogenic properties of HTLV-1. A large body of data witness the involvement of Tax-1 in the initiation of the oncogenic process mainly through a subversion of key pathways of cell activation and proliferation such as the NFkB pathway (21). The involvement of Tax-1 in tumorogenicity has received important biological support from Tax-1 transgenic mice which indeed develop leukemia and lymphomas (22). However, Tax-1 is not expressed in about 50% of ATL patients (23) and this has been taken as an argument to state the Tax-1 involvement in the initiation but not in the maintenance of the oncogenic process. More recent studies have partially challenged this notion since it has been found that even in Tax-1 negative ATL, at least in those in which there is no structural alteration of the *tax* gene, discrete bursts of Tax-1 expression in a minority of cells take place and may favor the growth of the ATL population (24). As opposed to Tax-1, HBZ is constantly expressed during infection and particularly in all ATL cases, where its presence seems to be involved in the maintenance of the cancer status, as witnessed also by experimental evidence in HBZ transgenic mice that develop leukemic/lymphomatous lesions (25).

## Expression and Subcellular Localization of Endogenous HBZ Protein

The alteration of cell homeostasis resulting in host pathology requires a careful analysis not only of the possible interactions of Tax-1 and HBZ with key molecules of cell host but also in a clear definition of the subcellular localization in which such interactions take place during the history of HTLV-1 infection. This has been particularly crucial for HBZ because most of its supposed biochemical and functional correlates of action were extrapolated by studies of HBZ-transfected cells mostly of non T cell origin (26). This was due to the lack of specific reagents that could detect endogenous HBZ in HTLV-1 infected cells or in ATL cells. The difficulty was overcome by the generation in my laboratory of a potent anti-HBZ monoclonal antibody, 4D4-F3, that can be used both in immunoprecipitation, western blotting and, importantly, in immunofluorescence and confocal microscopy (27). We then had a tool to analyze the expression, the molecular interaction with host cell factors and subcellular localization of HBZ in its natural contest.

One of the important aspects was the quantification of endogenous HBZ in its “physiological” setting compared to the one observed in classical transfection experiments. Indeed we could determine that the amount of HBZ measured in leukemic or HTLV-1-infected established cell lines was on the order of 17 to 40 thousand molecules per cell, and this estimate was at least 20 to 50 fold less than the amount found in 293T cells transfected with HBZ (27). This had an impact also in the assessment and the definition of the extent of previously described interactions between HBZ and cellular factors. While for example CBP and JunD could be shown to interact with endogenous HBZ in established chronically infected or leukemic cell lines, this interaction was barely visible in fresh leukemic cells from patients. This was paralleled by a faint and minor colocalization of endogenous HBZ with the above factors as well as with p300 and CREB-2 factors in fresh leukemic cells as compared to established cell lines, as assessed by confocal microscopy (27). Conversely, all the above factors strongly reacted biochemically and colocalized extensively with HBZ in 293T cells transfected with the HBZ cDNA. Thus, functional correlates of HBZ interaction with cellular factors drawn on the basis of HBZ expressed in transfected cells should be carefully re-evaluated on the basis of the real concentration of the endogenous viral protein expressed in its natural context, that is fresh HTLV-1 infected or ATL patient cells.

The second crucial aspect of HBZ that could be re-evaluated was its endogenous subcellular localization through the history of the infection, from initial infection to development of HTLV-1 associated pathologies. Initial studies based on HBZ transfection in cells, often not representative of the natural target of HTLV-1, suggested an exclusive nuclear localization of the viral protein (12) (26). Preliminary investigation by using our anti-HBZ monoclonal antibody confirmed the nuclear localization in established cell lines including the leukemic ATL-2 cells and one sample of fresh leukemic cells from an ATL patient (27). However, when we extended the analysis to fresh PBMC from HTLV-1-infected asymptomatic carriers (AC) we found instead an exclusive cytoplasmic localization of HBZ (28). Moreover, an exclusive cytoplasmic localization was also found in PBMC from HAM/TSP patients (28–30). All together, these findings led us to conclude that the unprecedented localization of HBZ in the cytoplasm could be considered as a marker of pathological distinction of HAM/TSP with respect to ATL (29). The extended analysis of AC and HAM/TSP revealed additional peculiarities of HBZ expression and subcellular localization. The percentage of HBZ-positive cells in PBMC of AC, as detected by accurate confocal microscopy, varied between 0 to 4%, significantly lower than the percentage of Tax-1 positive cells which varied between 0 to 11%. Interestingly, only in very few cases of AC we have been able to find the co-expression of HBZ and Tax-1 within the same cells (28, 29). Thus it appears that in PBMC of AC there is a sort of mutual exclusion of endogenous HBZ and Tax-1 expression at single cell level. The reasons of this apparent dichotomy require further investigation. Furthermore, when Tax-1 was expressed, it localized both in the cytoplasm and nucleus. In PBMC of HAM/TSP patients, the situation was very similar to AC with the distinctive difference of the higher percentage of both HBZ and Tax-1 positive cells, particularly of HBZ-positive cells (up to 10%), and this partially correlated with the increased proviral load observed in these patients (28). The fact that both in AC and HAM/TSP patients HBZ was localized exclusively in the cytoplasm led us to investigate whether this unprecedented subcellular localization was the result of a preferential shuttling of the viral protein in this compartment. If this were the case, treatment with leptomycin B (LMB), a drug inhibiting the CRM-1-depedent nuclear export (31), should result in preferential segregation of HBZ in the nucleus. However this was not the case, as treatment with LMB did not modify at all the cytoplasmic localization of HBZ in either AC or HAM/TSP. This result strongly suggested that HBZ was actively retained in the cytoplasm, possibly by a factor or factors whose molecular nature and intimate mechanism are at present actively investigated. In this respect it should be noted that it has been recently reported that in HBZ-transfected Jurkat T cells the viral protein may partially segregate in the cytoplasm as result of interaction with the T cell-specific protein THEMIS (32). This prompted us to analyze whether in PBMC of AC and HAM/TSP patients endogenous HBZ could interact and colocalize with THEMIS. By confocal microscopy, we found partial co-expression and colocalization of HBZ and THEMIS in certain cells but not in others where HBZ could be expressed in absence of THEMIS (28). Thus, expression of THEMIS is not a prerequisite for the expression and cytoplasmic localization of endogenous HBZ. Additional factors may intervene whose molecular nature and intimate mechanism are at present elusive.

## Endogenous HBZ in ATL

The above summarized studies, although mostly phenotypic in nature, did have an important impact on the previously assumed idea that HBZ was a nuclear protein and, as such, would exert its biological functions in the nucleus only. The change in paradigm generated by our results thus modified the perspective in the evolution of HTLV-1-associated pathology, strongly discriminating the HTLV-1-associated chronic inflammatory pathologies, such as HAM/TSP, from the cancer state such as ATL. Nevertheless, the unambiguous and exclusive cytoplasmic expression found in asymptomatic carriers, mostly representing early stages of HTLV-1 infection, compared with the nuclear localization in ATL cell lines, representing a very late effect of HTVL-1 infection, suggested that development of ATL could be accompanied by, and/or be associated to, a unidirectional cytoplasmic-to-nuclear transition of HBZ. If this were the case, we should have been able to find leukemic patients with a dual cytoplasmic and nuclear localization of the viral protein. In collaboration with the group of Olivier Hermine we thus investigated fresh leukemic cells of an extended number of both acute and chronic ATL patients, by immunofluorescence and confocal microscopy. With our surprise, we found that all patients analyzed, irrespective of the acute or chronic disease state, expressed HBZ in the cytoplasm and in the nucleus (33). Indeed, in most cases cytoplasmic expression was even more pronounced than nuclear expression. These data confirmed our hypothesis that ATL was accompanied by a subcellular cytoplasmic/nuclear relocalization of HBZ. Apparently therefore we filled the gap between the exclusive cytoplasmic HBZ localization found in AC and HAM/TSP patients and the exclusive HBZ nuclear localization mainly found previously in ATL cell lines and in very few ATL patients. Several additional interesting findings were observed in the extended analysis of fresh leukemic cells from patients that were rather unexpected. For example a relatively high proportion of HBZ-positive cells (up to 68%) were found in chronic ATL patients, a clinical state usually characterized by a low number of leukemic cells (34). As these patients did not progress toward an acute state, it is possible that this high number reflects the chronically HTLV-1 infected state of these patients on top of which leukemogenesis develops.

The other very interesting finding related to the study of fresh leukemic cells consisted in the evidence that treatment of the cells with LMB did not result in increased nuclear retention of HBZ. While in the case of AC and HAM/TSP a similar finding simply indicated that HBZ was not freely shuttling between cytoplasm and nucleus, in the case of leukemic cells cytoplasmic HBZ did not freely shuttle to the nucleus in presence, however, of a distinctive proportion of HBZ molecules already present in the nucleus. This suggests that the progressive nuclear translocation of HBZ takes place under a very strict control. The molecular mechanism(s) at the basis of the HBZ retention in the cytoplasm during the history of HTLV-1 infection from AS to the development of ATL are presently unknown. Oncogenic transformation is accompanied and caused mostly by accumulation of mutations that affect cell homeostasis and control of cell growth (35–37). If the factor(s) that retain HBZ in the cytoplasm are structurally mutated in ATL and cannot further exert their docking function, it can be expected that HBZ can freely migrate into the nucleus and thus the cytoplasmic component should be retained into the nucleus after LMB treatment. As this is not the case, the mechanism of active retention in the cytoplasm of part of the HBZ pool should be different. In search of additional candidates, beside the above described possibility that THEMIS might partially contribute to HBZ cytoplasmic retention, we analyzed a possible involvement of calreticulin as it has been shown that calreticulin acts as cytoplasmic retention molecule for Tax-1. By colocalizing and interacting with Tax-1, calreticulin regulates the shuttling between nucleus and cytoplasm of the viral molecule (38). However, in ATL patients HBZ was not clearly confined in calreticulin subcellular compartment, while Tax-1 partially did (33).

**Figure 1:**
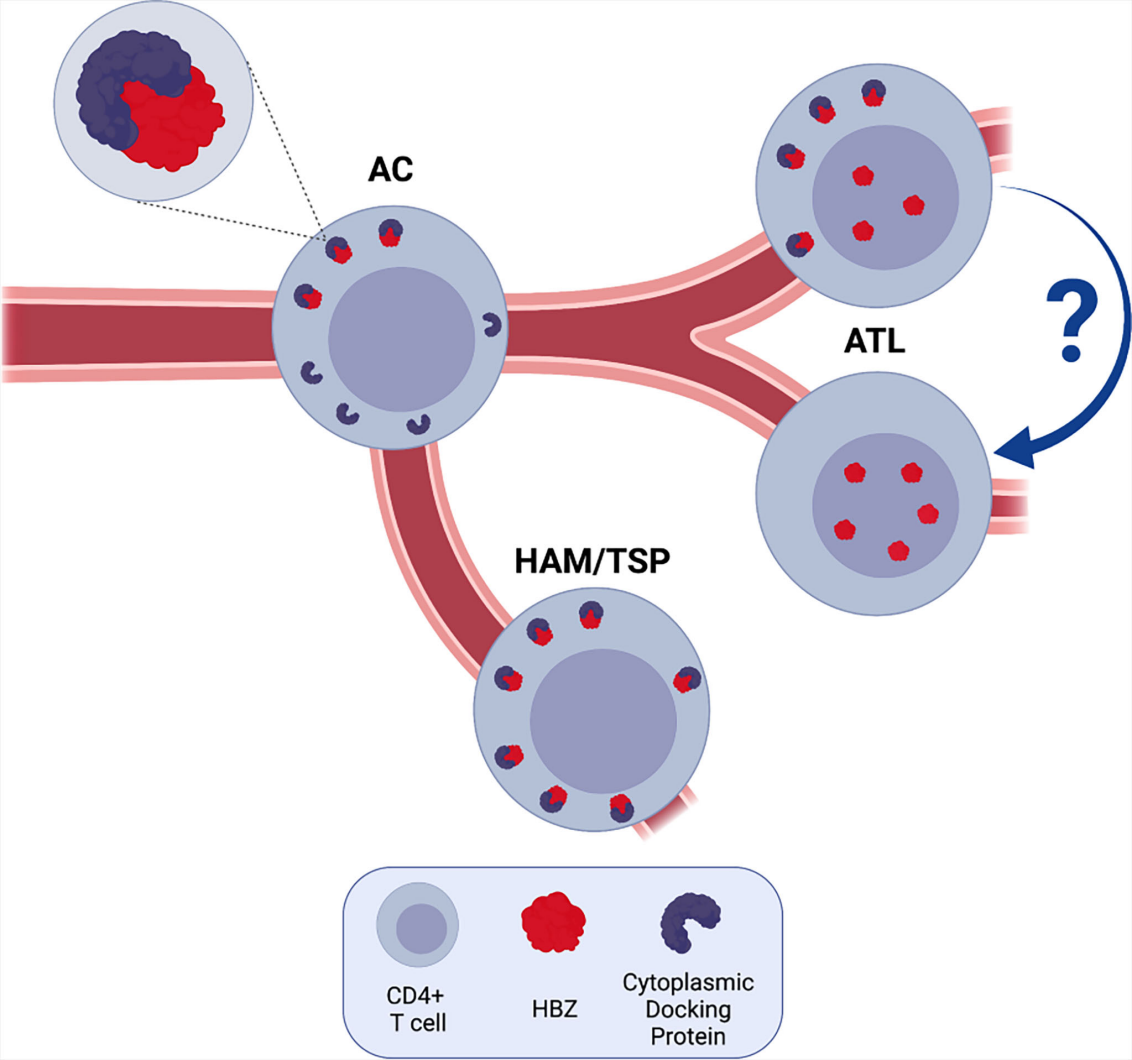
HBZ and the road to HTLV-1-mediated ATL.The HTLV-1-encoded HBZ protein (red symbol) is expressed in all phases of HTLV-1 infection to development of Adult T cell Leukemia (ATL). In CD4+ T cells of infected asymptomatic carriers (AC) and patients with HAM/TSP, HBZ resides exclusively in the cytoplasm and cannot migrate into the nucleus. For this reason, it is hypothesized that cytoplasmic docking factor(s) (blue symbol) actively retain the viral protein in this subcellular compartment. Development of leukemic state is accompanied by, or even partially dependent on, the progressive displacement of HBZ into the nucleus possibly because of a downregulation of docking factor expression or other still unknown mechanisms. The fact that certain leukemic patients express HBZ only in the nucleus may indicate a distinct pathway or a further evolution (arrow with a question mark) in the developmental history of ATL.

It may be conceivable that the process of cytoplasmic retention of HBZ would gradually diminish because of defects in the regulation of expression of gene (s) encoding still unknown docking factor(s). Lowering the amount of these molecules would allow HBZ to escape the interaction with them and freely migrate into the nucleus (**Figure 1**).

Another interesting point of our recent study was related to the expression of spliced versus unspliced HBZ mRNA in the fresh leukemic cells of ATL patients. The unprecedented HBZ cytoplasmic localization in ATL cells was paralleled by a more abundant expression of spliced vs unspliced form of HBZ mRNA (33), similarly to what we found in HAM/TSP patients and in AC that have exclusive cytoplasmic localization of HBZ protein (28, 29). Conversely, the ATL-2 leukemic cell line and leukemic cells of a previously described patient displaying a predominant HBZ nuclear localization, showed a more abundant or similar unspliced vs spliced HBZ (33), suggesting a possible correlation between subcellular protein localization and distinct forms of alternatively spliced HBZ mRNA.

## Future Directions

Research on the function of HTLV-1-encoded HBZ is rapidly evolving and certainly will witness important developments in the future. Many aspect of the HBZ biology are still incompletely clarified. Among them, two are of particular relevance in my opinion. First, the elucidation of the functional correlates of HBZ in the different subcellular compartments in which it localizes. As highlighted in this report, the unidirectional cytoplasmic-to-nuclear transition of HBZ protein, marking the passage from infection to development of leukemic state, underlines the importance of assessing the biological role of cytoplasmic HBZ in ATL. Importantly, the interaction of HBZ with host factors in the cytoplasm and in the nucleus which, I anticipate, will be more complex than previously envisaged, should better clarify the role of HBZ in the onset and persistence of the oncogenic process. In this direction, the study of the HBZ interactome initiated by several groups, including ours, is unveiling unexpected interactions with a large array of proteins involved, for example, in RNA splicing and stability (39), mechanisms to the core of gene expression, often altered in cancer.

Second, the full clarification of the role of HBZ mRNA in the HTLV-1 associated diseases. It has been previously suggested that HBZ mRNA plays a role in supporting proliferation of ATL cells (16). The rather unexpected nuclear compartmentalization of HBZ mRNA (40), confirmed and extended in a recent investigation (41) gives further support to the notion that HBZ mRNA is acting as a transcriptional modulator not only of the viral genome, where it may participate to viral latency (42), but also of host gene expression (16, 41, 43). Within this frame, HBZ mRNA behaves therefore as a bifunctional molecule with coding capacity and regulatory properties (44). It remains to be established the relative impact of HBZ mRNA, both in quantitative and temporal terms, to the process of oncogenesis vis-à-vis of HBZ protein.

## Author Contributions

RA conceived and wrote the paper. The author confirms being the sole contributor of this work and has approved it for publication.

## Funding

This work was supported by The Institutional Grant, University of Insubria, to RA; in part by European Community FP7 Grant no. 602893 “Cancer Vaccine Development for Hepatocellular Carcinoma-HepaVAC” http://www.hepavac.eu; and in part by Associazione Italiana per la Ricerca sul Cancro (AIRC) 2021, Grant no. 26195 “Novel anti-glioblastoma therapeutic vaccines based on optimal activation of tumor-specific CD4+ T helper cells”.

## Conflict of Interest

The author declares that the research was conducted in the absence of any commercial or financial relationships that could be construed as a potential conflict of interest.

## Publisher’s Note

All claims expressed in this article are solely those of the authors and do not necessarily represent those of their affiliated organizations, or those of the publisher, the editors and the reviewers. Any product that may be evaluated in this article, or claim that may be made by its manufacturer, is not guaranteed or endorsed by the publisher.
